# Allergen immunotherapy during the COVID‐19 pandemic—A survey of the German Society for Allergy and Clinical Immunology

**DOI:** 10.1002/clt2.12134

**Published:** 2022-03-28

**Authors:** Oliver Pfaar, Eckard Hamelmann, Ludger Klimek, Christian Taube, Christian Vogelberg, Martin Wagenmann, Thomas Werfel, Margitta Worm

**Affiliations:** ^1^ Department of Otorhinolaryngology Head and Neck Surgery Section of Rhinology and Allergy University Hospital Marburg Philipps‐Universität Marburg Marburg Germany; ^2^ Department of Paediatrics Children's Center Bethel University Bielefeld Bielefeld Germany; ^3^ Center for Rhinology and Allergology Wiesbaden Germany; ^4^ Department of Pulmonary Medicine University Medicine Essen ‐ Ruhrlandklinik Essen Germany; ^5^ Department of Pediatric Pulmonology and Allergy University Hospital Carl Gustav Carus Technical University of Dresden Dresden Germany; ^6^ Department of Otorhinolaryngology (HNO‐Klinik) Düsseldorf University Hospital (UKD) Düsseldorf Germany; ^7^ Department of Dermatoloy and Allergy Medizinische Hochschule Hannover Hannover Germany; ^8^ Division of Allergology and Immunology, Department Dermatology Venerology and Allergology Charité Universitätsmedizin Berlin Berlin Germany

**Keywords:** allergen immunotherapy, COVID‐19, pandemic, safety, SARS‐CoV‐2, survey

## Abstract

**Background:**

When the coronavirus pandemic 2019 (COVID‐19) emerged, concerns were also raised regarding the safety of allergen immunotherapy (AIT). The German Society for Allergology and Clinical Immunology (DGAKI) conducted a survey to collect real‐world data on the daily routine of administering subcutaneous AIT (SCIT) and sublingual AIT (SLIT) during the COVID‐19 pandemic.

**Methods:**

A web‐based retrospective survey using the online platform *survio* with 26 standardized questions was used to survey physicians treating allergic patients during the pandemic.

**Results:**

Three hundred and forty‐five physicians who regularly offer and perform AIT in German‐speaking countries responded to the questions. 70.4% of the respondents stated that they regularly initiated and dosed up SCIT for inhalant allergies (41.4% venom‐SCIT, 73.6% SLIT), and 85.2% of the respondents stated that they continued SCIT for inhalant allergies during the maintenance phase in a regular way (59.1% venom‐SCIT, 90.4% SLIT) in healthy patients without current symptoms indicating an infection with COVID‐19. With regard to tolerability, there was no evidence for increased occurrence of adverse events in patients without current symptoms of COVID‐19 infection during the pandemic.

**Conclusions:**

This retrospective study demonstrated adherence to national and international position papers of AIT during the COVID‐19 pandemic in German‐speaking countries. Besides, the survey has confirmed a good tolerability of AIT for both SCIT and SLIT.

**Abbreviations**
AAAAI: American Academy of Allergy and Clinical Immunology
AIT: allergen immunotherapy
ARC: allergic rhinoconjunctivitis
ARIA: allergic rhinitis and its impact on asthma
COPD: chronic obstructive pulmonary disease
COVID‐19: Coronavirus disease 2019
DGAKI: German Society for Allergology and Clinical Immunology
EAACI: European Academy of Allergy and Clinical Immunology
ENT: ear nose throat
SARS‐CoV‐2: severe acute respiratory syndrome coronavirus 2
SCIT: Subcutaneous immunotherapy
SLIT: Sublingual immunotherapy
WHO: World Health Organisation

## 1 INTRODUCTION

In December 2019, a novel strain of human coronavirus, the severe acute respiratory syndrome coronavirus 2 (SARS‐CoV‐2), was identified as the cause of the Coronavirus disease 2019 (COVID‐19) declared to be pandemic in March, 2020.^1^, ^2^, ^3^, ^4^ Despite the fact that a variety of viral and bacterial infections are known to trigger or aggravate exacerbations in asthmatic patients, initial analyses did not show an increased risk for severe courses of SARS‐CoV‐2 infections in allergic patients.^5^, ^6^, ^7^ However, data were limited and inconclusive at the beginning of the pandemic. The European Academy of Allergy and Clinical Immunology (EAACI), but also national societies, therefore published several position papers,^8^, ^9^, ^10^ how to manage optimal care of allergic patients and allergen immunotherapy (AIT) during the so called “first wave” of the pandemic.

AIT is a disease‐modifying treatment option for various allergic diseases and can be administered subcutaneously (SCIT) or sublingually (SLIT).^11^, ^12^, ^13^, ^14^, ^15^ It provides long‐term benefits, if adherence is ensured.^16^, ^17^ Though AIT generally is a safe and effective treatment option in allergic diseases, uncertainties regarding the safety of this treatment arose in the context of the COVID‐19 pandemic. Among these, treatment providers struggled to prioritize face‐to‐face encounters considering the recommendation to avoid social contact.^18^ However, continuation of therapy is generally recommended in the aforementioned international position papers^8^, ^9^, ^10^ as well as in the previously adapted national versions for German‐speaking countries.^19^, ^20^ Therefore, a triage for example, via telephone should be performed to identify patients with symptoms of COVID‐19 prior to consultation in order to minimize the risk of infection. Initiation of SCIT or SLIT in patients, without known COVID‐19 infection or symptoms indicating such, is generally possible according to the recommendations adapted to German‐speaking countries.^19^, ^20^ However, a thorough history and examination for signs of infection at the start of treatment and at each subsequent SCIT injection or SLIT administration is recommended.^19^ Regarding continuation of AIT, SCIT in particular, should be continued, especially for potentially life‐threatening allergies such as insect venom allergy. Lengthening the injection intervals may be considered. Termination of SLIT is also unlikely to be necessary.^19^, ^20^

However, it remains unclear whether continued AIT in the setting of COVID‐19 infection is safe and data are lacking. In general, interruption of AIT is indicated when viral infections occur, therefore experts recommend discontinuing AIT in case of COVID‐19 infection as well.^8^, ^9^, ^20^, ^21^

Based on the international consensus, EAACI previously conducted a survey to analyse the situation in different countries worldwide regarding the implementation of AIT in routine clinical practice.^18^ Since national position and consensus papers for German‐speaking countries have followed, the German Society for Allergology and Clinical Immunology (DGAKI) conducted the present survey based on the international EAACI study with a special focus on the German‐speaking countries Germany, Austria and Switzerland. The aim of the survey was to determine adherence of the practitioners to the published recommendations and to obtain further information on practical aspects and the general tolerability of AIT during the pandemic. Based on these data, valuable conclusions can be drawn regarding measures to manage AIT in this or in further potential pandemics in the future.

## 2 METHODS

Twnty‐six questions regarding the practical implementation in the AIT routine and the specific tolerability in the context of the COVID 19 pandemic were elaborated. This questionnaire was then formally approved by the DGAKI and made available to German‐speaking doctors, predominantly based in Germany, Austria and Switzerland via the online platform *survio* (*Survio* s.r.o. Brünn*, Czech Republik*) between July 6th 2020 and February 27th 2021.

The questions can generally be categorized in four domains. The first domain (Questions 1–11) includes basic information on the respondent and his or her patients. The second domain (Questions 12–21) addresses management and tolerability of AIT in patients without current symptoms indicating COVID‐19 infection. The third domain (Questions 22–25) addresses management of AIT in patients despite (early) symptoms of COVID‐19 infection and/or positive test. The last domain contains an additional question, asking respondents to rate five statements under the assumption of a “second wave” in autumn/winter.

This survey has been registered at the Institutional Review Board of the Medical School of Philipps‐University Marburg, Germany, and complies with the current International Conference on Harmonization E‐6 Guidelines for Good Clinical Practice (ICH‐GCP‐E6).

## 3 RESULTS

### 3.1 Domain 1, Questions 1–11

In the present survey, 345 physicians from German‐speaking countries participated. Among the respondents, 42.3% were DGAKI members, 1.4% DGAKI junior members, and 56.2% non‐DGAKI members. Most physicians responding to the questionnaire were practicing in Germany (95.4%), 2.9% in Austria, 1.2% in Switzerland, and 0.6% in other countries. Most participating doctors worked in private practices (80.9%), followed by university hospitals (13.9%), municipal hospitals (2.9%), private clinics (0.3%) and others (4.3%). Most of the respondents treated both pediatric and adult allergic patients (70.4%), followed by doctors treating mainly adult patients (22.3%) and doctors treating only children (7.2%). In terms of specialties, ear nose throat (ENT) physicians were the largest responding speciality with 68.3%, followed by dermatologists (18%), pulmonologists (6.7%), paediatricians (6.1%), and internists (4.1%). Overall 47.4% of the respondents reported being allergists (allergy is a subspecialty in Germany).

Most respondents had more than 10 years of experience in performing AIT. Physicians were asked how many percent of their patients with allergic rhinoconjunctivitis (ARC), asthma, or both concurrent conditions received AIT. Regarding ARC, 53,8% reported treating 50%–80% of their allergic patients with AIT. Similar results were obtained for patients with asthma and in cases of concomitant ARC and asthma. Furthermore, respondents were asked to indicate how many of their allergic patients with ARC received SLIT or SCIT in percentage (in total 100%). The majority of respondents indicated that they treated more patients with SCIT than with SLIT.

It was further examined whether the practitioners were aware of position papers for conducting AIT during the pandemic. In total, 72.5% of respondents stated that those were available, 8.7% indicated no available position papers and 18.8% did not know if those were available. Among all respondents, only 8.7% reported following national or international position papers or other recommendations when conducting AIT during the COVID‐19 pandemic. However, 68.3% reported following a similar strategy prior to publication of the position papers. 9.3% of respondents followed an alternative strategy (Table 1).

**TABLE 1:** Management of Allergen Immunotherapy practice during the COVID‐19 pandemic (Questions 9–11)

	Responses (*n* = 345)	%
Question 9. Are there any national consensus or position papers for the management of AIT during the COVID‐19 pandemic available in your country?		
Yes	250	72.5
No	30	8.7
I Don't know	65	18.8
Question 10. Do you follow any national or international (e.g., EAACI, WHO, AAAAI) position paper/Consensus reports for the management of AIT during the COVID‐19 pandemic?		
I don't know.	47	13.7
No, we're following a different strategy.	32	9.3
Yes, but we followed a similar strategy prior to knowing about the position papers.	235	68.3
Yes	30	8.7
Question 11. Which measures did you perform during the COVID‐19 lockdown or during pandemic‐related hardest restrictions for the management of your allergic patients?		
Stop both first and follow‐up consultations	6	1.7
Replace face‐to‐face visits by phone calls for all patients	7	2
Replace face‐to‐face visits by phone calls for follow‐up, but to maintain face‐to‐face visits for new patients	9	2.6
Maintain face‐to‐face visits for all patients	255	73.9
Other	68	19.7

Abbreviations: AAAAI, American Academy of Allergy and Clinical Immunology; AIT, allergen immunotherapy; EAACI, European Academy of Allergy and Clinical Immunology; WHO, World Health Organisation.

Respondents were further asked to specify how they had practiced care of their allergic patients during lockdown. Most of the respondents (73.9%) stated that they maintained regular in person follow‐up consultations. 19.7% followed an individual strategy, for example, continuation of therapy that has previously been initiated but no further treatment initiations. 2.6% of the respondents reported to have completely suspended follow‐up treatments by replacing them by telephone consultations but have continued to perform initial treatments on site. Both initial and follow‐up treatments were completely replaced by telephone consultations by 2.0% of respondents, while 1.7% indicated they had completely suspended initial and follow‐up treatments (Table 1).

### 3.2 Domain 2, Questions 12–21

Physicians were questioned regarding their strategy concerning AIT in patients without signs of COVID‐19 infection. Regarding SCIT, regular up‐dosing phase was performed by 70.4% of the respondents. 16.2% did not initiate SCIT and planned to postpone the initiation until after the pandemic. Another 5.5% reported SCIT‐initiation with a modified therapeutic scheme (e.g., fewer visits for the up‐dosing phase). 1.7% of the respondents decided to switch from SCIT to SLIT. In case of patients receiving SCIT for venom allergies, 41.4% of the respondents decided to perform regular treatment schedule, while 13.0% postponed treatment. 2.6% of respondents initiated SCIT, but modified the therapy regimen (e.g., shorter inpatient up‐dose). 42.9% reported other. Most of these respondents did not have any requests for SCIT for venom allergy during the pandemic or they did not perform this treatment in the first place. Regarding SLIT, 73.6% of respondents started therapy under regular circumstances, whereas 12.2% reported delayed initiation. 2.0% of the respondents initiated therapy with modified up‐dosing. 12.2% disclosed via commentary function not to perform SLIT (Table 2).

**TABLE 2:** Initiation of AIT in patients without symptoms to suspect COVID‐19 (Questions 12–14)

Question 12. For patients receiving SCIT (without signs of COVID‐19 infection) please select the applied option for initiation of AIT during the pandemic (during the COVID‐19 lockdown or during pandemic‐related hardest restrictions for the management of your allergic patients).		
Not to initiate, but to postpone the initiation to a time point after the pandemic	56	16.2
To initiate, but amend the up dosing schedule	19	5.5
To initiate as planned under regular circumstances	243	70.4
To initiate SLIT as alternative application route and self‐ administration	6	1.7
Other	21	6.1
Question 13. For patients receiving SCIT for venom allergies (bee/wasp venom) (without signs of COVID‐19 infection), please select the applied option for the initiation during the pandemic (during the COVID‐19 lockdown or during pandemic‐related hardest restrictions for the management of your allergic patients).		
Not to initiate, but to postpone the initiation to a time point after the pandemic	45	13
To initiate, but amend the up dosing schedule	9	2.6
To initiate as planned under regular circumstances	143	41.4
Other	148	42.9
Question 14. For patients receiving SLIT (without signs of COVID‐19 infection), please select the applied option for the initiation during the pandemic (during the COVID‐19 lockdown or during pandemic‐related hardest restrictions for the management of your allergic patients).		
Not to initiate, but to postpone the initiation to a time point after the pandemic	42	12.2
To initiate, but modified the up‐dosing schedule	7	2
To initiate as planned under regular circumstances	254	73.6
Other	42	12.2

Abbreviations: AIT, allergen immunotherapy; SCIT, subcutaneous immunotherapy; SLIT, sublingual immunotherapy.

Furthermore, physicians were asked about their approach regarding the continuation of AIT in patients without evidence of COVID‐19 infection. For patients who were in the maintenance phase, 85.2% of the respondents reported to have continued SCIT on a regular basis. 9.6% continued SCIT but increased the time intervals between applications. 0.9% reported discontinuing SCIT and postponing therapy. 0.3% reported switching SCIT to SLIT. For patients with venom‐allergies, 59.1% of the respondents continued SCIT regularly. 6.1% continued SCIT but prolonged application intervals and 1.2% discontinued treatment. In case of patients receiving SLIT in the maintenance phase 90.4% of the respondents reported continuing treatment regularly, while 0.9% continued SLIT under dose‐reduction‐schedule. 0.6% discontinued SLIT and delayed treatment.

Regarding patients without evidence of COVID‐19 infection who were in the initiation phase of SCIT, 95.7% of the respondents reported good tolerability, while 15 respondents (4.3%) reported the occurrence of adverse events. Among patients who were in the initiation phase of SLIT (without evidence of COVID‐19), 89.9% of respondents reported good tolerability, whereas 35 respondents (10.1%) reported the occurrence of adverse events. In patients without current symptoms of possible COVID‐19 infection who were in the maintenance phase, 98% and 93.6% of respondents reported good tolerance of SCIT and SLIT, respectively (Table 3).

**TABLE 3:** Adverse events of AIT in patients without symptoms to suspect COVID‐19 (initiation and maintenance) (Questions 18–21).

Question 18: For patients receiving SCIT in the initiation period:		
SCIT was well tolerated	330	95.7
SCIT lead to significant adverse event	15	4.3
Question 19: For patients receiving SLIT in the initiation period:		
SLIT was well tolerated	310	89.9
SLIT lead to significant adverse event	35	10.1
Question 20: For patients receiving SCIT in the maintenance period:		
SCIT was well tolerated	338	98
SCIT lead to significant adverse event	7	2
Question 21: For patients receiving SLIT in the maintenance period:		
SLIT was well tolerated	323	93.6
SLIT lead to significant adverse event	22	6.4

Abbreviations: SCIT, subcutaneous immunotherapy; SLIT, sublingual immunotherapy.

#### 3.2.1 Domain 3, Questions 22–25

Interview participants were asked to indicate whether patients experienced COVID‐19 infection during treatment. For patients in the initiation phase of SCIT, 9 respondents (10%) indicated that patients experienced early symptoms of COVID‐19 infection. Furthermore, 10 respondents (11.1%) reported that patients received a positive COVID‐19 test result. Regarding the initiation phase of SLIT, 7.1% of respondents reported early symptoms of COVID‐19 infection in patients and 6.0% reported that patients received a positive COVID‐19 test result. Question 24 has been formulated out of the context and was therefore excluded from the analysis. For patients who received SLIT in the maintenance phase, 5 (6.1%) of the respondents reported early symptoms of COVID‐19 infection in their patients and 7 (8.5%) reported positive tests for COVID‐19 infection for patients in the maintenance phase of SLIT.

### 3.3 Domain 4

Respondents were asked to rate statements assuming a “second wave” in autumn/winter 2020. The statements were to be rated on a scale from 0 to 5. 0 corresponded to “I disagree” to 5 ″I agree to the fullest”. The first statement was “*In general, AIT should be paused because the risk of adverse side effects of AIT poses an unacceptable risk to patients*.” 83.5% of the respondents disagreed (by giving “0” points). The second statement was “*In general, AIT should be paused as the risk of infection with SARS‐COV 2 (by doctors/medical staff or other patients) poses an unacceptable risk to patients*.” 71% of the respondents disagreed (by giving “0” points). The third statement was “*In general, AIT should only be performed in patients with a negative test result for SARS‐COV 2*.” 56.2% of the respondents disagreed (by giving “0” points). The fourth statement was “*In general, AIT should be changed from SCIT to SLIT*.” 70.7% of the respondents disagreed (by giving “0” points). The fifth statement was “*In general, AIT should 72 only be performed in specialized centres/outpatient clinics*,” and 62.9% of the respondents disagreed (by giving “0” points).

## 4 DISCUSSION

This report is based on a previous EAACI international survey on the practical aspects and safety of AIT in the context of the COVID 19 pandemic.^18^ As the EAACI/Allergic Rhinitis and Its Impact on Asthma (ARIA) position paper on the management of AIT during the pandemic has been adapted to the national situation in German‐speaking countries,^19^, ^20^ this survey aims to provide an overview of the impact of these position papers on daily practice. In addition, this study is intended to determine compliance with national recommendations on AIT. 345 physicians participated in this DGAKI survey, most of whom are based in Germany, followed by Austria and Switzerland.

The previous international survey referred to above found that almost 50% of respondents reported a lack of academic recommendations on AIT during the pandemic at national level. However, 41.91% felt that the available position papers were helpful and 38.15% stated following a similar strategy prior to becoming aware of those recommendations.^18^ In total about 80% of the interviewed physicians performed therapy of allergic patients in line with the recommendations in the international position paper. The authors of the survey have attributed this to the expertise and evidence‐based approach of physicians performing AIT.^18^, ^22^

In the current survey in the German‐speaking countries, most respondents (72.5%) indicated that position papers were available at the national level. Even though only 8.7% stated following these, 68.3% indicated already following a similar strategy before getting aware of the recommendations. Hence, a total of 77% carried out the therapy in accordance to the “gold standard” as recommended in the position papers. These findings further underline the assumption of the EAACI survey, that physicians, performing AIT, are well‐trained and experienced in this treatment option.

The international position papers^9^ as well as those adapted for German‐speaking countries^19^, ^20^ contain directives intended to guarantee a good quality of treatment. On the other hand, concepts have been developed to ensure the safety of patients and healthcare providers. For example, a triage via telephone prior to consultation was recommended in order to check the necessity and to minimize the risk of transmitting infections to other patients or medical staff. Furthermore, the use of telemedicine was encouraged to further minimize unnecessary contacts.^8^, ^9^, ^19^, ^20^

Telemedicine has been shown to be effective in allergic patient care and is a promising option for optimizing patient care in times of social contact avoidance.^23^ However, in the present survey, the majority of respondents (73.9%) indicated maintaining face‐to‐face meetings. This finding is contrary to the results of the international survey, where 40% of the respondents stated that they had switched to telemedicine during the pandemic.^18^ On the one hand this result could be related to the fact that the respondents mainly performed SCIT, which usually requires face‐to‐face meetings. On the other hand, the respondents were mainly physicians in outpatient practices (80.9%), which supports findings in a survey among pneumologists in Germany on the perception of the COVID‐19 pandemic.^24^ This study revealed significant differences between participants from practitioners and clinicians regarding the perception of utility of telemedicine. Respondents from the outpatient sector attributed significantly lower relevance to telemedicine and utilized this opportunity less frequently, claiming regulatory restrictions.^24^

Restrictions in health care occurred worldwide throughout the pandemic. A decline was also seen in the care of allergic patients, as the EAACI survey revealed that only 10% of the respondents initiated SCIT as usual.^18^ On the other hand, 70.4% of the respondents carried out the initiation of AIT in a regular manner during the pandemic in the study presented in this article. These results are encouraging, as the authors of the international survey^18^ feared a severe grade of undertreatment due to the significant reduction in the initiation of AIT. However, the recommendation in German‐speaking countries is that the initiation of AIT can be safely performed in patients without signs of COVID‐19 infection,^19^, ^20^ and consequently the majority of practitioners seem to have followed this. However, in other countries, even more severely affected by the pandemic during the first wave of COVID‐19, the focus may have been set more on preserving resources and avoiding social contact in general. This has been indicated in a current consensus document of an expert panel in the US.^25^

With regard to AIT for insect venom allergy, the authors of the EAACI survey were particularly concerned, as 40% of respondents did not initiate it due to the pandemic.^18^ However, in the current survey presented here, only 13% of the respondents claimed to postpone treatment initiation. Based on these findings, practitioners may have prioritized treatment of this potentially life‐threatening disease based on current position papers as given above.

However, a study by Worm et al. surveyed allergy departments in Germany, Austria and Switzerland and compared the number of initiations of AIT for hymenoptera‐venom from March‐June 2019 to March‐June 2020. The authors indicated a decline of treatment initiations by 48.5%.^26^ The authors attributed this reduction to limited hospital capacity and the hesitation of patients to seek hospital care during the pandemic. The discrepancy between the results of the study by Worm et al. and the present survey could be explained by the differences in the studied population. As Worm et al. exclusively surveyed large allergy centres of university hospitals, 80% of the respondents in the present survey were physicians in outpatient practices. This could indicate that patients were more likely to visit practices during the pandemic. Moreover, outpatient practices might have been less affected by the pandemic in regard to capacity and resources. This assumption is supported by the results of the data from a survey among pneumologists.^24^ According to this analysis, respondents from university hospitals and maximum care hospitals as well as regional hospitals perceived significantly higher changes in their daily work due to the COVID‐19 pandemic than employees in the outpatient sector.^24^ However, in another recently published survey by the EAACI aimed to evaluate real‐life consequences on the COVID‐19 pandemic in allergy practices in general, only 60% of the respondents informed about not‐changing prescriptions in venom immunotherapy^27^ which again indicates to be below the results found in our survey.

With regard to maintenance phase of AIT during the pandemic, the present study showed encouraging results, analogous to the international data.^18^ 85.2% of the respondents continued SCIT as usual. For patients with insect venom allergy, SCIT was only discontinued by 1.2% of the respondents. SLIT was even reported to be continued regularly by 90.4% of physicians. Therefore, most respondents seem to have followed the national recommendations. Besides, our survey showed no unexpected increase of reporting of adverse events during the initiation and the maintenance phase of both SCIT and SLIT and confirmed the good safety profile of this treatment in healthy patients without current symptoms of a possible COVID‐19 infection as demonstrated in the international survey.^18^

The EAACI position paper and the German adaptation recommended discontinuation of AIT in patients with early signs of COVID‐19 infection.^9^, ^19^, ^20^ The presented survey revealed that early symptoms or a positive test‐result of COVID‐19 infection were apparent in a minority of patients. However, the answers were inconclusive if and to what extent adverse events became apparent under these circumstances.

At the end of this survey, respondents were asked to agree or disagree with statements regarding the continuation of AIT, assuming a second wave in autumn/winter. Most respondents disagreed with the statements that AIT should be paused during a second wave. Interestingly most respondents also disagreed that AIT should only be continued if patients were tested negative for COVID‐19‐infection. This corresponds to the results of the previously conducted international survey^18^ and can be explained by the good experience of the respondents with the continuation of AIT during the “*first wave*” in general.

Moreover, most respondents disagreed that SCIT should be switched to SLIT and most of the interviewees disagreed that AIT should only be performed in specialized centres or clinics. This contradicts the EAACI survey. In the latter, about 50% agreed with this statement.^18^ These results are not particularly surprising in respect of the population of physicians examined. As already mentioned, mainly physicians in outpatient practices with broad experience in this therapy answered the present survey. As other analyses, the present study indicates that practices might have been less affected by the pandemic and/or patients preferred treatment in the outpatient setting.

## 5 CONCLUSION

Allergic diseases are highly prevalent and there is a significant degree of suffering among patients. It is important to maintain high quality care of allergic patients even during the COVID‐19 pandemic that also severely affected German‐speaking countries. First, this retrospective study demonstrates adherence to national and international position papers of AIT during the COVID‐19 pandemic in German‐speaking countries. Besides, the survey has not found evidence of reduced tolerability of AIT as the only proven immunomodulating therapy in atopic diseases in the context of the pandemic. The analysis indicates that AIT can be safely administered in patients without evidence of COVID 19 infection.

## CONFLICT OF INTERESTS

Dr. Pfaar reports grants and personal fees from ALK‐Abelló, grants and personal fees from Allergopharma, grants and personal fees from Stallergenes Greer, grants and personal fees from HAL Allergy Holding B.V./HAL Allergie GmbH, grants and personal fees from Bencard Allergie GmbH/Allergy Therapeutics, grants and personal fees from Lofarma, grants from Biomay, grants from Circassia, grants and personal fees from ASIT Biotech Tools S.A., grants and personal fees from Laboratorios LETI/LETI Pharma, personal fees from MEDA Pharma/MYLAN, grants and personal fees from Anergis S.A., personal fees from Mobile Chamber Experts (a GA2LEN Partner), personal fees from Indoor Biotechnologies, grants and personal fees from GlaxoSmithKline, personal fees from Astellas Pharma Global, personal fees from EUFOREA, personal fees from ROXALL Medizin, personal fees from Novartis, personal fees from Sanofi‐Aventis and Sanofi‐Genzyme, personal fees from Med Update Europe GmbH, personal fees from streamedup! GmbH, grants from Pohl‐Boskamp, grants from Inmunotek S.L., personal fees from John Wiley and Sons, AS, personal fees from Paul‐Martini‐Stiftung (PMS), personal fees from Regeneron Pharmaceuticals Inc., personal fees from RG Aerztefortbildung, personal fees from Institut für Disease Management, personal fees from Springer GmbH, personal fees from AstraZeneca, personal fees from IQVIA Commercial, personal fees from Ingress Health, outside the submitted work; and member of EAACI Excom, member of ext. board of directors DGAKI; coordinator, main‐ or co‐author of different position papers and guidelines in rhinology, allergology and allergen‐immunotherapy. Dr. Hamelmann has nothing to disclose. Dr. Klimek reports grants and personal fees from Allergopharma, grants and personal fees from Viatris, personal fees from HAL Allergie, personal fees from ALK Abelló, grants and personal fees from LETI Pharma, grants and personal fees from Stallergenes, grants from Quintiles, grants and personal fees from Sanofi, grants from ASIT biotech, grants from Lofarma, personal fees from Allergy Therapeut., grants from AstraZeneca, grants and personal fees from GSK, grants from Inmunotek, personal fees from Cassella med, personal fees from Novartis, personal fees from Regeneron Pharmaceuticals, personal fees from ROXALL Medizin GmbH, outside the submitted work; and Membership: AeDA; DGHNO; Deutsche Akademie für Allergologie und klinische Immunologie; HNO‐BV; GPA; EAACI. Dr. Taube has nothing to disclose. Dr. Vogelberg has nothing to disclose. Dr. Wagenmann reports grants and personal fees from ALK‐Abelló, personal fees from Allergopharma, grants and personal fees from AstraZeneca, personal fees from Bencard, personal fees from Genzyme, grants and personal fees from GSK, personal fees from Infectopharm, personal fees from LETI Pharma, personal fees from med update, grants and personal fees from Novartis, grants and personal fees from Sanofi Aventis, grants from Takeda, personal fees from Stallergenes, outside the submitted work.Dr. Werfel has nothing to disclose. Margitta Worm has served as a speaker, consultant or PI for ALK, Abbvie, Pfizer, Stallergenes, Allergopharma, Aimmune, Thermo Fisher,Novartis, EliLilly, Sanofi, Regeneron, Leo Pharma and Viatris.

## AUTHOR CONTRIBUTIONS

**Oliver Pfaar:** Conceptualization; Data curation; Investigation; Methodology; Project administration; Supervision; Validation; Writing—original draft; Writing—review & editing. **Eckard Hamelmann:** Conceptualization; Data curation; Funding acquisition; Investigation; Methodology; Supervision; Writing—review & editing. **Ludger Klimek:** Conceptualization; Methodology; Supervision; Writing—review & editing. **Christian Taube:** Conceptualization; Funding acquisition; Project administration; Supervision; Writing—review & editing. **Christian Vogelberg:** Conceptualization; Investigation; Methodology; Writing—review & editing. **Martin Wagenmann:** Conceptualization; Funding acquisition; Investigation; Methodology; Supervision; Writing—review & editing. **Thomas Werfel:** Conceptualization; Funding acquisition; Supervision; Writing—review & editing. **Margitta Worm:** Conceptualization; Funding acquisition; Project administration; Supervision; Writing—review & editing.
